# Exposure of *Mycobacterium tuberculosis* to human alveolar lining fluid shows temporal and strain-specific adaptation to the lung environment

**DOI:** 10.3389/ftubr.2024.1352806

**Published:** 2024-03-04

**Authors:** Anna Allué-Guardia, Andreu Garcia-Vilanova, Alyssa M. Schami, Angélica M. Olmo-Fontánez, Amberlee Hicks, Jay Peters, Diego J. Maselli, Mark D. Wewers, Yufeng Wang, Jordi B. Torrelles

**Affiliations:** ^1^Population Health Program, Tuberculosis Group, Texas Biomedical Research Institute, San Antonio, TX, United States; ^2^Integrated Biomedical Sciences Program, University of Texas Health Science Center at San Antonio, San Antonio, TX, United States; ^3^Division of Pulmonary and Critical Care Medicine, School of Medicine, UT Health San Antonio, San Antonio, TX, United States; ^4^Department of Internal Medicine, Pulmonary, Critical Care and Sleep Medicine Division, College of Medicine, The Ohio State University, Columbus, OH, United States; ^5^Department of Molecular Microbiology and Immunology, South Texas Center for Emerging Infectious Diseases, University of Texas at San Antonio, San Antonio, TX, United States; ^6^International Center for the Advancement of Research and Education (I•CARE), Texas Biomedical Research Institute, San Antonio, TX, United States

**Keywords:** *Mycobacterium tuberculosis*, alveolar lining fluid, lung mucosa, transcriptomics, cell envelope

## Abstract

Upon infection, *Mycobacterium tuberculosis* (*M.tb*) reaches the alveolar space and comes in close contact with the lung mucosa or human alveolar lining fluid (ALF) for an uncertain period of time prior to its encounter with alveolar cells. We showed that homeostatic ALF hydrolytic enzymes modify the *M.tb* cell envelope, driving *M.tb*-host cell interactions. Still, the contribution of ALF during *M.tb* infection is poorly understood. Here, we exposed 4 *M.tb* strains with different levels of virulence, transmissibility, and drug resistance (DR) to physiological concentrations of human ALF for 15-min and 12-h, and performed RNA sequencing. Gene expression analysis showed a temporal and strain-specific adaptation to human ALF. Differential expression (DE) of ALF-exposed *vs*. unexposed *M.tb* revealed a total of 397 DE genes associated with lipid metabolism, cell envelope and processes, intermediary metabolism and respiration, and regulatory proteins, among others. Most DE genes were detected at 12-h post-ALF exposure, with DR-*M.tb* strain W-7642 having the highest number of DE genes. Interestingly, genes from the KstR2 regulon, which controls the degradation of cholesterol C and D rings, were significantly upregulated in all strains post-ALF exposure. These results indicate that *M.tb*-ALF contact drives initial bacterial metabolic and physiologic changes, which may have implications in the early events of *M.tb* infection.

## Introduction

Airborne pathogen *Mycobacterium tuberculosis* (*M.tb*) is the causative agent of tuberculosis (TB), one of the top leading causes of death worldwide due to a single infectious agent, with 11 million cases and ~1.6 million attributed deaths in 2021 and many underreported diagnoses due to the coronavirus disease 2019 (COVID-19) pandemic disruptions in global healthcare (1). Indeed, a 20% raise in the number of TB deaths is predicted over the next 5 years, resulting in a setback of at least 5 to 8 years in End-TB Strategy (2, 3). Further, relocation of resources and access limitations to healthcare during the COVID-19 pandemic predict an increase in the number of drug-resistant (DR) TB cases worldwide (4).

During the process of natural infection, *M.tb* is initially deposited in the lung alveolar space where it first encounters the lung mucosa, composed of a surfactant lipid layer and an aqueous hypophase (called alveolar lining fluid or ALF). *M.tb* is thought to be in close contact with ALF for an undefined period (from min to h) before encountering host alveolar resident cells and after its escape from dying cells during the initial phases of infection, where it can interact with ALF innate soluble components (5). Specifically, homeostatic hydrolytic activities (hydrolases) present in human ALF, whose main function is ALF degradation and recycling, alter the *M.tb* cell envelope upon ALF-*M.tb* exposure in as little as 15 min (6). These ALF-induced cell envelope alterations in *M.tb* are shown to influence subsequent interactions with immune host cells *in vitro* and *in vivo*, ultimately determining the infection outcome (6–12). Indeed, exposure to ALF resulted in decreased association and intracellular growth of *M.tb* in human macrophages, as well as increased *M.tb* intracellular killing in neutrophils while limiting tissue damage, overall leading to a better control of the infection by professional phagocytes (6, 8). In addition to ALF hydrolases, levels and functionality of other ALF soluble components are significantly altered in some human populations such as the elderly, resulting in accelerated *M.tb* growth *in vitro* (human macrophages and alveolar epithelial cells) and exacerbated infection *in vivo* in young mice when *M.tb* is exposed to elderly ALF (10, 12, 13). Thus, the host ALF status and functionality might play an important role in shaping the *M.tb* cell envelope during the initial stages of the infection (10, 13). However, the overall impact of the ALF on the adaptation of *M.tb* to the alveolar space prior to being recognized by host cells and subsequent infection is still largely unknown.

Biochemical studies have also determined that the *M.tb* cell envelope composition is quite diverse between phylogenetic lineages and/or geographical regions, and even among strains with different drug resistance patterns (14–17), which can lead to different interactions with host immune cells (18). Still, it is uncertain if different *M.tb* strains/lineages exposed to human ALF will have distinct metabolic responses prior to encountering host cells.

In this study, we aimed to determine the overall and strain-specific effects of human ALF exposure on different *M.tb* genomic backgrounds driving *M.tb*-host interactions (6–12). For this, we selected four well-characterized *M.tb* strains, two from lineage 4 (H_37_R_v_ and CDC1551, European, American, and African) and two from lineage 2 (HN878 and W-7642, Beijing/East-Asian). Besides their lineage, *M.tb* strains were also selected based on their levels of transmissibility, virulence, and drug resistance. H_37_R_v_ is a common reference laboratory strain derived from a patient with chronic pulmonary TB at the beginning of the 20th century and selected for its virulence in animal models (19). CDC1551 is a clinical strain isolated from a TB outbreak in the mid-90s in a small rural area close to the Kentucky-Tennessee border. This strain is considered of low virulence but highly transmissible in humans (20). Though highly immunogenic at early stages of infection, CDC1551 is efficiently controlled in the lungs of infected animals (21). Strain HN878 was isolated during a TB outbreak in Houston, Texas, during the late 90s (22). Its hypervirulent phenotype is attributed to the presence of cell envelope phenolic glycolipids (PGLs) and triglycerides, and is associated with a rapid bacterial growth and reduced survival due to decreased TH1 host protective immunity driving cavitary disease (23). Finally, W-7642 was isolated in the early 90s after a series of outbreaks in New York City caused by the spread of a multidrug resistant (MDR)-*M.tb* Beijing clone. This strain is also considered highly virulent, since it caused increased mortality rates when compared to other clinical isolates (24).

Thus, in order to define the metabolic adaptations of these four *M.tb* strains to the human ALF environment, we performed RNA sequencing after their exposure to human ALF, and determined shared and strain-specific differentially expressed genes between strains when compared to unexposed bacteria (Supplementary Figure S1). Our results indicate that time-dependent exposure of *M.tb* to ALF drives global metabolic and physiologic changes in *M.tb* with potential implications in bacterial recognition by host cells and the establishment and outcome of the infection.

## Materials and methods

### Human subjects and ethics statement

Blood and bronchoalveolar lavage fluid (BALF) was collected from healthy adult volunteers, in strict accordance with the US Code of Federal Regulations and approved Local Regulations (The Ohio State University Human Subjects IRB numbers 2008H0135 & 2008H0119 and Texas Biomedical Research Institute/UT-Health San Antonio Human Subjects IRB numbers HSC20170667H & HSC20170315H), and Good Clinical Practice as approved by the National Institutes of Health (NIAID/DMID branch), with written informed consent from all human subjects. Healthy adult volunteers (18–45 years old) were TST- or IGRA-negative and were recruited from both sexes (50:50 male:female ratio) with no discrimination based on race or ethnicity. The following comorbidities were excluded: drug and alcohol users, smokers, asthma, acute pneumonia, upper/lower respiratory tract infections, acute illness/chronic condition, heart disease, diabetes, obesity, obstructive pulmonary disease (COPD), renal failure, liver failure, hepatitis, thyroid disease, rheumatoid arthritis, immunosuppression or taking nonsteroidal anti-inflammatory agents, human immunodeficiency virus (HIV)/AIDS, cancer requiring chemotherapy, leukemia/lymphoma, seizure history, blood disorders, depression, lidocaine allergies (used during the BAL procedure), pregnancy, nontuberculous mycobacterial infection, and TB.

### Collection of BALF and ALF isolation

BALF was collected from healthy adult donors in 80 mL of sterile endotoxin-free human isotonic saline (0.9% NaCl), filtered through 0.2 μm filters, and concentrated 20-fold using Amicon Ultra Centrifugal Filter Units with a 10-kDa molecular mass cutoff membrane (Millipore Sigma, Burlington, MA, USA) to obtain the ALF physiological concentration within the lung (1 mg/mL of phospholipid), as we described (6–13, 25). We use the *in vivo* relevant levels of 0.5 to 1.5 mg/ml of surfactant phospholipids in the lung mucosa (26), measured by phospholipid assay (27). The 20-fold concentration process removes free surfactant lipids and free fatty acids of <10-kDa, leaving functional hydrolases and other proteins such as surfactant proteins A and D (SP-A/SP-D), complement, and antimicrobial peptides in the ALF fraction, as well as other molecules of >10-kDa such as cholesterol. Effective lipid removal (<10-kDa) from ALF was confirmed in previous studies by mass spectrometry (MS) of fatty acid methyl ester (FAMES) derivatives (6). ALF was then aliquoted and stored at −80°C until further use. Samples were maintained at 4°C on ice and in endotoxin-free sterile conditions during the whole procedure.

### Bacterial cultures and ALF exposure

*M.tb* strains CDC1551, H_37_R_v_ (ATCC# 25618), HN878 (BEI Resources, NR-13647), and W-7642 were cultured from frozen stocks in 7H11 agar plates (BD BBL), supplemented with oleic acid, albumin, dextrose and catalase (OADC) for 14 days at 37°C. Single bacterial suspensions in 0.9% NaCl were obtained as described (6–12, 25), and bacterial pellets (~ 1 x 10^9^ bacteria/ml) obtained after centrifugation (13,000 x *g*, 10 min) were further incubated in a 50 μl pool of ALFs (mixture of ALFs from *n* = 17 different donors in equal proportions), 50:50 male:female ratio, no discrimination in ethnicity and race) for 15 min or ~12 h at 37°C (no agitation). After exposure, ALF was removed and bacterial pellets washed with 0.9% NaCl. Pellets were further incubated in RNAProtect (Qiagen, Hilden, Germany) for 10 min at room temperature (RT), centrifuged at 13,000 × *g* and stored at −80°C until further use. For each of the strains, unexposed bacteria were processed in parallel and used as controls (bacterial pellets incubated for 15 min or ~12 h at 37°C without ALF). Two independent experiments were performed: two different exposures to the same pool of ALFs on different days, using single bacterial suspensions of newly fresh harvested bacteria grown in 7H11 agar for each strain studied.

### RNA extraction, library prep and RNA sequencing

Stored bacterial pellets were used for RNA extraction using the Quick-RNA Fungal/Bacterial Miniprep kit (Zymo Research, Irvine, CA, USA), following the manufacturer's protocol. Briefly, bacterial pellet was resuspended in lysis buffer and transferred to a ZR BashingBead Lysis tube containing 0.1 mm and 0.5 mm ceramic beads. A bead beating procedure to break the mycobacterial cell envelope was performed in a BioSpec Mini-Beadbeater-24 (six cycles of 45 s at 3,800 rpm, with 3 min intervals on a cooler rack). RNA in the supernatant was isolated using Zymo-spin columns, with an in-column DNAse I treatment, and eluted in nuclease-free water. A second DNAse treatment was performed on the isolated RNA using the TURBO DNAse reagent (Thermo Fisher Scientific, Waltham, MA, USA), and RNA was further purified using the RNA Clean & Concentrator kit (Zymo Research, Irvine, CA, USA). RNA final concentration was measured with a Qubit 4 Fluorometer using the HS RNA kit (Thermo Fisher Scientific, Waltham, MA, USA), and RIN numbers were obtained using the 4200 TapeStation System (Agilent, Santa Clara, CA, USA) to determine RNA quality before sequencing. A stranded whole transcriptome RNA library was constructed using the Zymo-Seq RiboFree Total RNA kit (Zymo Research) following the manufacturer's guidelines. RNA (500 ng) was used as input to construct the library, which included a rRNA depletion step of 30 min followed by 14 cycles of PCR. A 75-bp paired-end (PE) sequencing was conducted using a NextSeq 500 Mid Output Illumina platform, obtaining 5–8 million PE reads per sample. All samples (from *n* = 2 independent experiments) were sequenced in the same flow cell to avoid sequencing batch effects.

### Data analysis

Raw reads were trimmed (phred score of 30) and aligned to the reference genome H_37_R_v_ (Genbank NC_000962.3) (28) using STAR v2.5.3a with default values in the Partek Flow Genomic Analysis software (v9.0.20.0202, Partek Inc., Chesterfield, MO, USA). Further analyses were performed in the iDEP.94 software (http://bioinformatics.sdstate.edu/idep94/) (29): first, uploaded read count raw data were filtered (0.5 CPM, *n* = 1 library) to remove lowly expressed genes and transformed with edgeR as log_2_ (CPM + c) using default settings (30), and used for clustering and principal component Analysis (PCA). Hierarchical clustering of normalized gene expression levels was calculated using default values (correlation distance, average linkage, cutoff Z score of 4), and shown as a heatmap. Then, significant differentially expressed genes (DEGs) between ALF-exposed *M.tb* and unexposed *M.tb* (control) for each of the strains and time points were identified using the DESeq2 method (31) in iDEP.94, with an FDR cutoff of 0.1 and a minimum fold change (FC) of 2 (corresponding to a log_2_ FC equal or greater than an absolute value of 1). The Benjamini-Hochberg procedure was used to adjust the false discovery rate (FDR), and fold changes were calculated using an Empirical Bayes shrinkage approach (31) and the Wald test implemented in DeSeq2. Functional categories for DEGs were extracted from Mycobrowser (32). Gene enrichment analysis was performed in ShinyGO 0.77 using default settings (FDR cutoff of 0.05 and minimum pathway size of 2, Pathway database: all available gene sets) (33). A summary of the experimental conditions and workflow used for RNA sequencing and data analysis is shown in Supplementary Figure S1.

### Validation of gene expression by RT-qPCR

Some of the genes that were differentially expressed were selected for validation using RT-qPCR (Supplementary Table S1). Selection criteria was based on genes discussed more in detail in the manuscript, belonging to different pathways and/or categories, and that showed significant differences between strains and/or time points (*echA20, KstR2, mymA, papA1, papA3, mmpL10, mmpL8, mprA, sigF, espC* and *esxH*). Strains selected for RT-qPCR where the ones that showed the most differential expression for each of the genes. Briefly, 500 ng of RNA (same RNA that was used for RNA-seq) were used for the synthesis of cDNA using the RevertAid H Minus First Strand cDNA Synthesis kit (Thermo Fisher Scientific, Waltham, MA, USA) with random hexamer primers, following the manufacturer's instructions. Then, cDNA and specific primers were used in a 20 μl qPCR reaction with PowerUp SYBR Green Master Mix (Applied Biosystems, Waltham, MA, USA) run in an Applied Biosystems 7500 Real-Time PCR instrument with the following settings: reporter SYBR Green, no quencher, passive reference dye ROX, standard ramp speed, and continuous melt curve ramp increment, as described (25). Relative expression was calculated using the 2^−Δ*ΔCT*^ method (34) with *sigA* as the housekeeping gene.

### Statistical analyses

Statistical significance for bacterial uptake and intracellular growth rate was determined by multiple paired Student's *t* tests (ALF-exposed *vs*. unexposed *M.tb*). A 2-way ANOVA for multiple comparisons with an uncorrected Fisher's LSD test and main effects model was used for qPCR data (ΔCt ALF-exposed *vs*. ΔCt unexposed *M.tb* for each of the strains and time points).

## Results

### Alignment statistics, PCA, and clustering of samples

RNA from four *M.tb* strains (H_37_R_v_, CDC1551, HN878, and W-7642) exposed to human ALF for 15-min and 12-h was extracted and sequenced. The total average number of reads/sample after removing low quality reads during the initial quality control (QC) was 6.15 million, ranging from ~ 4.1 to 11.3 million. The number of aligned reads to the *M.tb* reference genome was ~ 90% on average. Samples with <70% of aligned reads were analyzed using Kraken (35) to discard potential sample contamination (data not shown). All samples had a coverage of more than 97% of their genome after alignment, with GC content of 62%. A Pearson correlation matrix was computed, showing a correlation of more than r = 0.97 between sample replicates (Supplementary Figure S2A). Our quality post-alignment statistics and quality control metrics are found in Supplementary Table S2.

**Figure 1:**
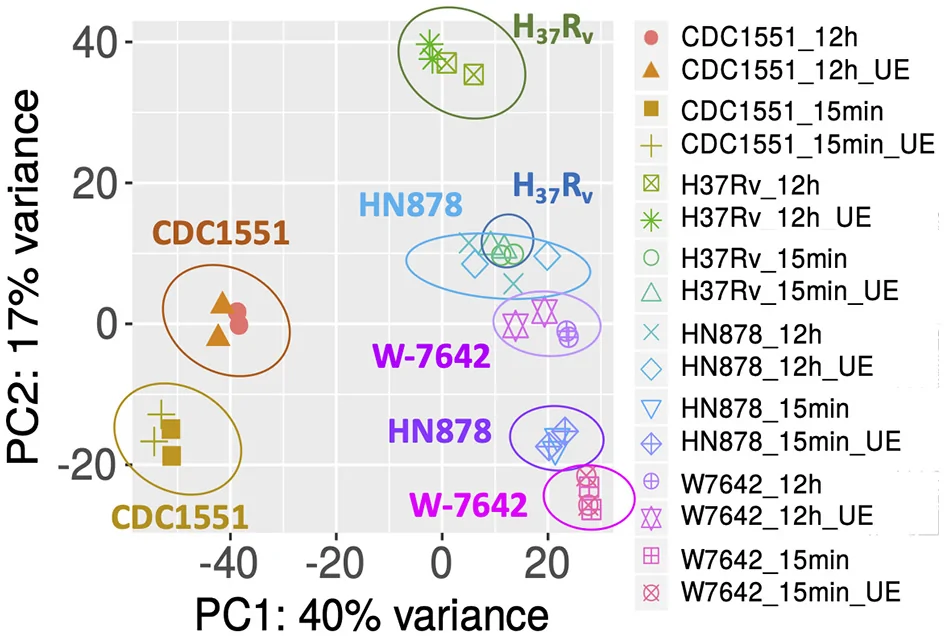
PCA of gene expression data of samples analyzed in this study: four *M.tb* strains (CDC1551, H_37_R_v_, HN878, W-7642), each exposed to a human ALF pool at its physiological concentration or unexposed (UE) for 15-min or 12-h, as detailed in the Methods. Replicates (*n* = 2) are shown with the same symbol and/or color. Axes show the variation in gene expression by each major Principal Component (PC1 and PC2). Graph was generated in iDEP.94.

Raw aligned data was then transformed and used for sample clustering and Principal Component Analysis (PCA). PCA grouped the samples based on *M.tb* strain (CDC1551, H_37_R_v_, HN878, W-7642) and incubation time (15-min and 12-h), the main sources of variance in this dataset, indicating temporal and strain-specific changes in transcriptional profiles of *M.tb* both before and after ALF exposure (Figure 1). Strain CDC1551 was furthest from the others at both time points tested, suggesting a more distinct transcriptional profile regardless of ALF exposure. We note here that the exposure time (15-min *vs*. 12-h) seems to have a bigger effect on the strains' transcriptome than the ALF exposure itself, since ALF-exposed and unexposed conditions are closely grouped in all the strains and time points. This indicates that the act of going from agar plates to being in suspension is causing a great number of transcriptional changes in *M.tb*, as one would expect. For this reason, unexposed bacteria was processed in parallel for each of the time points and used as control/baseline, and differential expression analyses were focused on the effects of ALF exposure (ALF-exposed *vs*. unexposed *M.tb*) at each time point rather than comparing exposure times.

Hierarchical clustering of normalized gene expression classified the samples in four main clusters (Supplementary Figure S2B). Cluster 1 (HN878 and W-7642 at 15-min) was closest to cluster 2 (HN878 and W-7642 at 12-h), indicating that those two strains presented the most similar transcriptional profile at each of the time points tested. They were followed by H_37_R_v_ at both time points (cluster 3) and CDC1551 (cluster 4), furthest from the others.

### Functional categories and enrichment of differentially expressed genes (DEGs)

**Figure 2:**
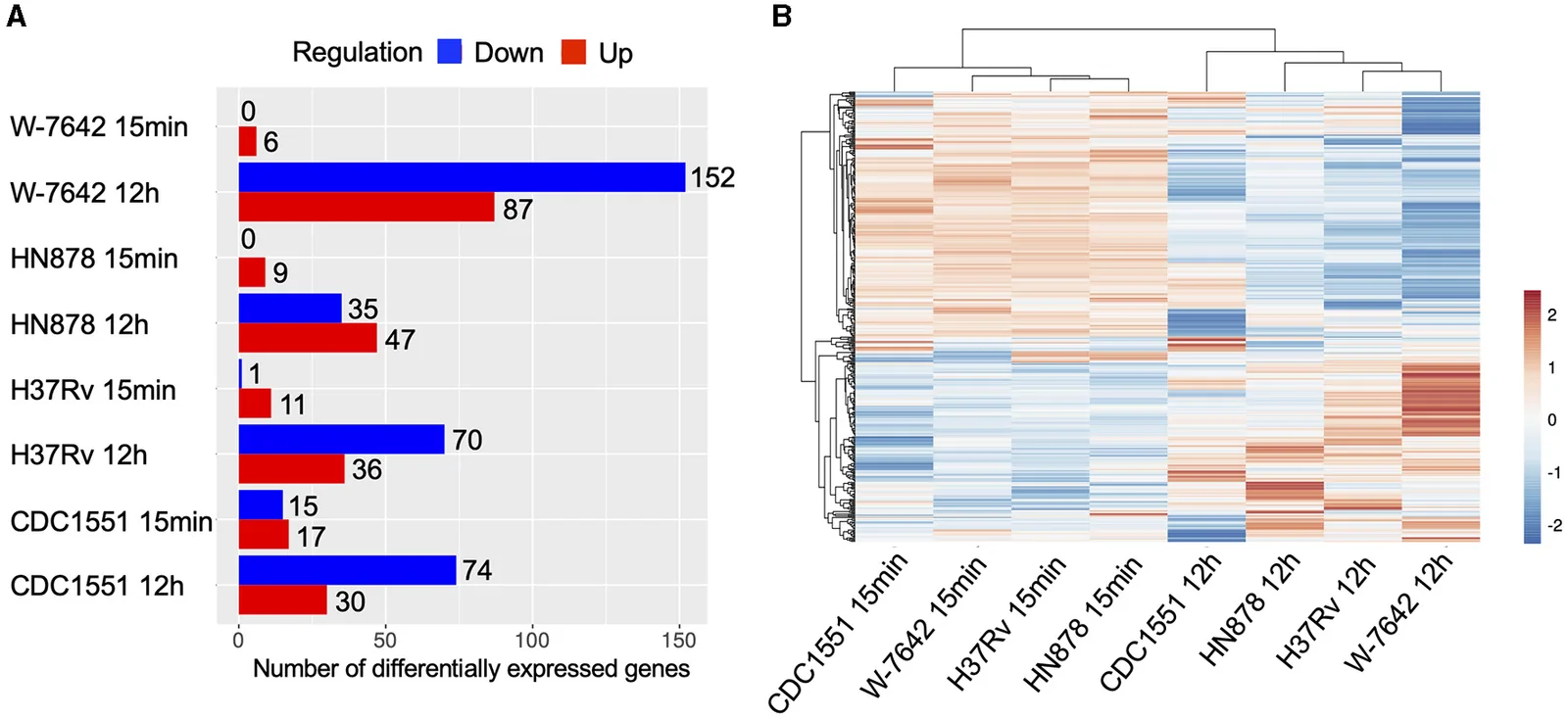
Differential expression analysis (ALF-exposed *M.tb vs*. unexposed *M.tb*) was performed for each of the strains (CDC1551, H_37_R_v_, HN878, W-7642) and time points (15-min and 12-h) using the DeSeq2 method in the iDEP.94 software, with the following settings: log_2_FC greater than an absolute value of 1, FDR <0.1. **(A)** Number of differentially expressed genes (upregulated in red, downregulated in blue) for each of the strains and time points. Graph was generated in iDEP.94. **(B)** A heatmap of DEGs was constructed using the log_2_FC values in ClustVis (https://biit.cs.ut.ee/clustvis/) (36) with default settings (average correlation clustering), showing separation of strains in two major clusters based on ALF exposure time. Upregulated genes: orange; downregulated genes: blue.

We performed DE analysis of ALF-exposed *vs*. unexposed *M.tb* for each of the strains at both time points. A total of 397 DEGs were identified, with DR-*M.tb* strain W-7642 being the one with the most transcriptional changes after exposure to ALF for 12-h (152 downregulated and 87 upregulated genes) compared to unexposed bacteria (Figure 2A). Exposure for 15-min to ALF resulted only in a few DEGs: 32 in CDC1551, 12 in H_37_R_v_, 9 in HN878, and 6 in W-7642. Hierarchical clustering using DEGs clearly classified our samples in two main clusters based on the ALF exposure time (15-min *vs*. 12-h) (Figure 2B), indicating a temporal adaptation to the lung mucosa, with a much greater number of DEGs at 12-h compared to 15-min.

**Figure 3:**
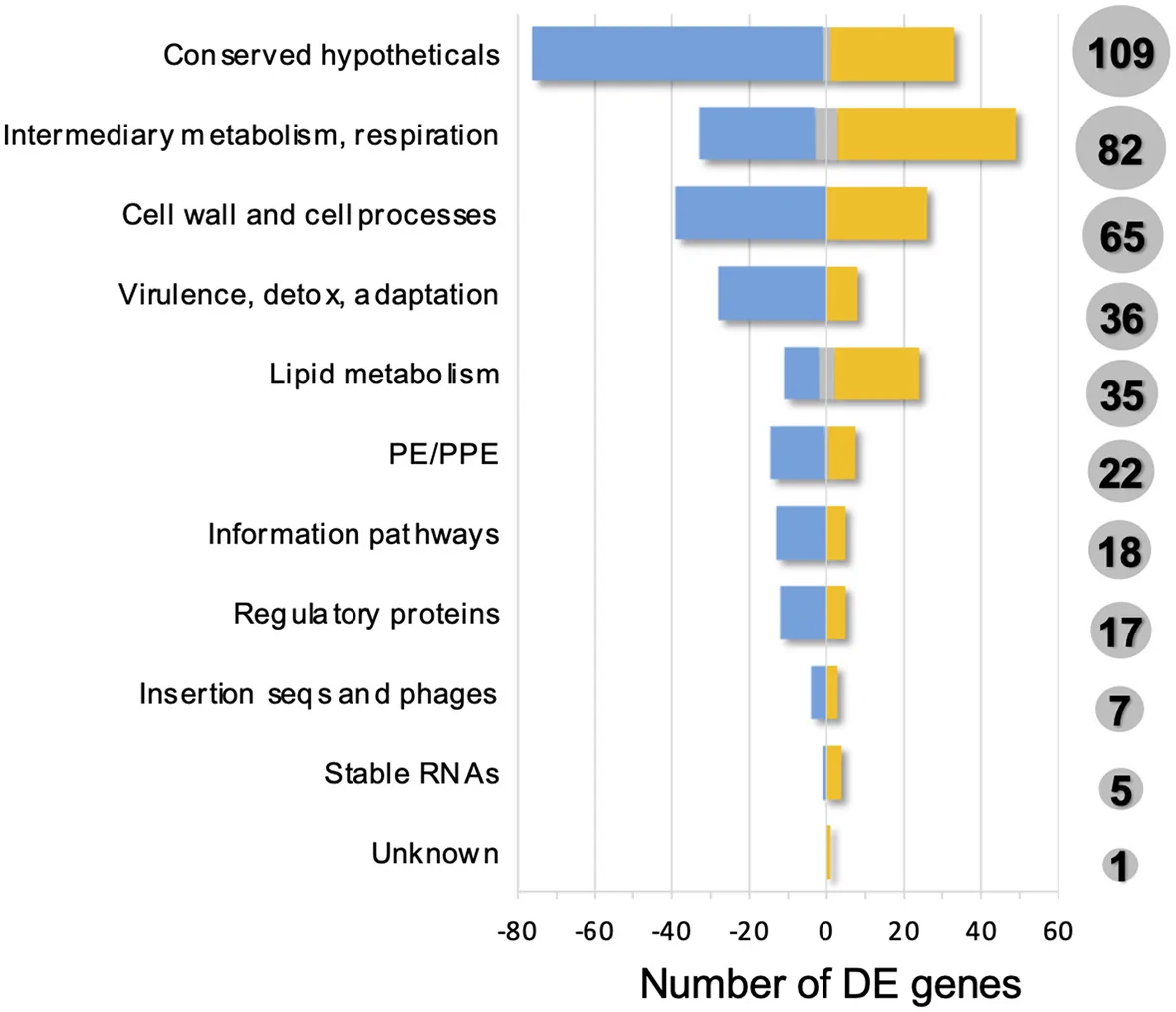
Functional categories of differentially expressed genes (ALF-exposed *M.tb vs*. unexposed *M.tb*) found in this study. A total of 397 DEGs were classified into 11 functional categories, as defined in Mycobrowser. Total number of DEGs are shown in gray circles for each functional category. Upregulated DEGs in one or more strains: yellow; downregulated DEGs in one or more strains: blue; up or downregulated DEGs depending on the strain: gray.

Identified DEGs belonged to different functional categories, including but not restricted to: conserved hypotheticals, intermediary metabolism and respiration, cell wall and cell processes, virulence, detoxification and adaptation, lipid metabolism, PE/PPE, information pathways, and regulatory proteins (Figure 3, Supplementary Table S3). At 15-min post-ALF exposure, the few DEGs found were associated to either lipid or intermediate metabolism, respiration, regulatory proteins or cell wall processes, except for strain CDC1551 which had more DEGs compared to the other strains and included other categories such as information pathways and PE/PPE (Supplementary Figure S3). At 12-h after ALF exposure, DEGs belonged to additional categories like virulence, detoxification and host adaptation, as well as insertion sequences and mobile genetic elements (MGEs) (Supplementary Figure S3).

**Figure 4:**
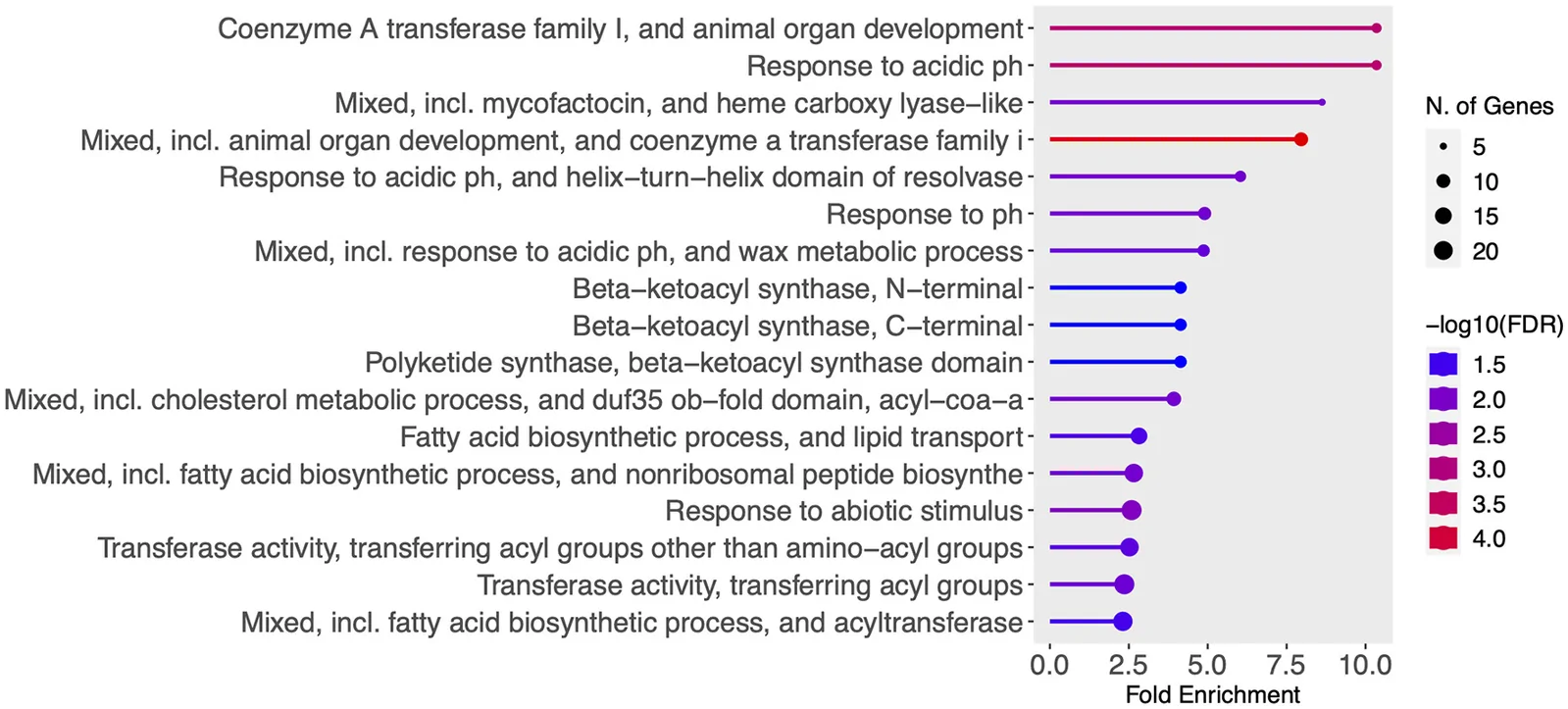
Gene enrichment analysis of all 397 identified DEGs was performed in ShinyGO 0.77 using default settings: FDR cutoff of 0.05, minimum pathway size of 2, Pathway database—all available gene sets. Significant enriched GO terms (FDR <0.05) were sorted by fold enrichment and are shown. Graph color depicts significance [-log10 (FDR)] and the number of DEGs belonging to each enriched pathway is shown in proportional circles.

We next performed gene enrichment analysis for the 397 total identified DEGs, and found 17 significantly enriched GO terms, including response to environmental pH, fatty acid biosynthetic process and lipid transport and associated beta-ketoacyl synthase activity, acyl transferase activity, cholesterol metabolic process, and mycofactocin and heme activity (Figure 4). Other enriched GO terms that did not pass the FDR cutoff of 0.05 were sulfolipid metabolic process, protein secretion by type VII secretion system, PPE superfamily, lipid biosynthesis, response to temperature and hypoxia, sigma factor activity, and phenolic phthiocerol biosynthetic process, among others (Supplementary Table S4).

### Exposure to ALF activates the KstR2 regulon associated with cholesterol utilization in *M.tb*

The cholesterol catabolic pathway is highly conserved in mycolic acid-containing actinomycetes, and is encoded by more than 100 genes in *M.tb* (37). There are three distinct sub-pathways or processes during cholesterol catabolism, according to the three parts of the molecule: side chain degradation, A & B ring degradation, and C & D ring degradation (38). Two major regulons, controlled by two TetR-type transcriptional regulators, have been associated with these processes: KstR1 regulates the transcription of side chain and A & B ring degradation genes, while KstR2 controls the transcription of C & D ring degradation genes (39). Other genes though to be regulated by cholesterol belong to the mammalian cell-entry Mce3R regulon, implicated in stress resistance (40), and the SigE regulon, essential for virulence (41).

**Figure 5:**
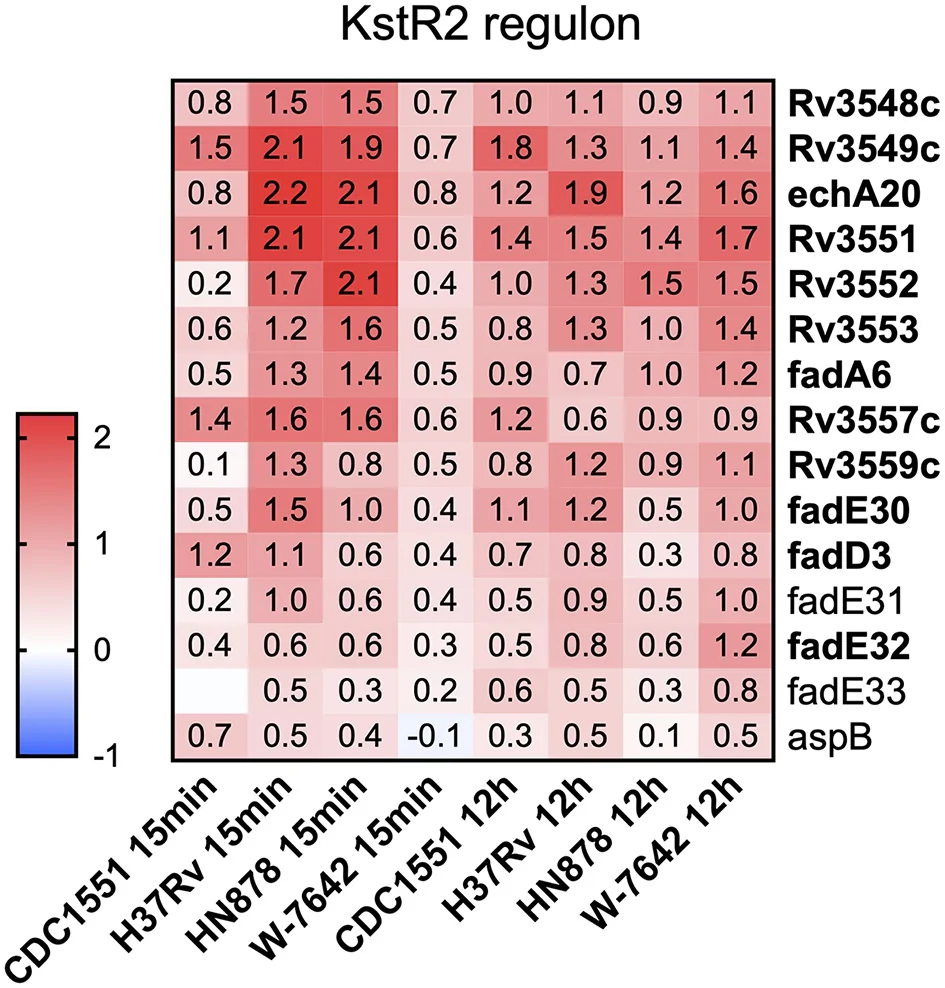
A heatmap of cholesterol utilization genes associated with the KstR2 regulon was generated in Graphpad Prism v9.1.1. Cells depict differential expression as log_2_FC values (ALF-exposed *M.tb vs*. unexposed bacteria) for each of the genes and strains/time points, upregulated: red, downregulated: blue. Genes in bold indicate significant DEGs (log_2_FC greater than an absolute value of 1, FDR <0.1) in one or more of the strains and/or time points.

Our results indicate that most KstR2 genes (12/15) were significantly upregulated (Log_2_FC >1 and FDR <0.1) either at 15-min or 12-h post-ALF exposure or both time points in one or more of the *M.tb* strains tested, with the exception of strain W-7642, where none of the KstR2 genes reached significant upregulation at 15-min (Figure 5, Supplementary Table S3). This included the genes for C & D ring degradation (3 out of 5 genes were significantly upregulated in at least one of the strains/conditions tested). In contrast, genes associated with the KstR1 regulon, including ATP-dependent cholesterol import genes encoded by the Mce4 transport system (42) and side chain and A&B ring degradation genes, were not differentially expressed when compared to unexposed *M.tb* at any of the ALF exposure periods of time tested (Supplementary Table S3). Only *ltp3* from the KstR1 regulon, encoding a keto acyl-CoA thiolase involved in the cholesterol side chain degradation (43), was significantly downregulated in strain CDC1551 at 12-h post-ALF exposure. Other significant DEGs associated with cholesterol side chain degradation were *fadE30, fadE32, echA20*, and *fadA6* (all upregulated and controlled by KstR2), and *fadB2* (downregulated in all four strains after 12-h of ALF exposure) (Supplementary Table S3). In addition, of the 23 genes associated with the Mce3R regulon, only two were significantly differentially expressed: *yrbE3B*, encoding a hypothetical membrane protein with phospholipid transport activity, was upregulated in strain HN878 at 12 h post-exposure, while *mce3F* was downregulated in H_37_R_v_ and W-7642 at that same time point (Supplementary Table S3). None of the cholesterol-associated *sigE* regulon genes were differentially expressed in our dataset under the experimental conditions tested.

### Exposure to ALF shows a temporal adaptation of *M.tb* to the lung mucosa

**Figure 6:**
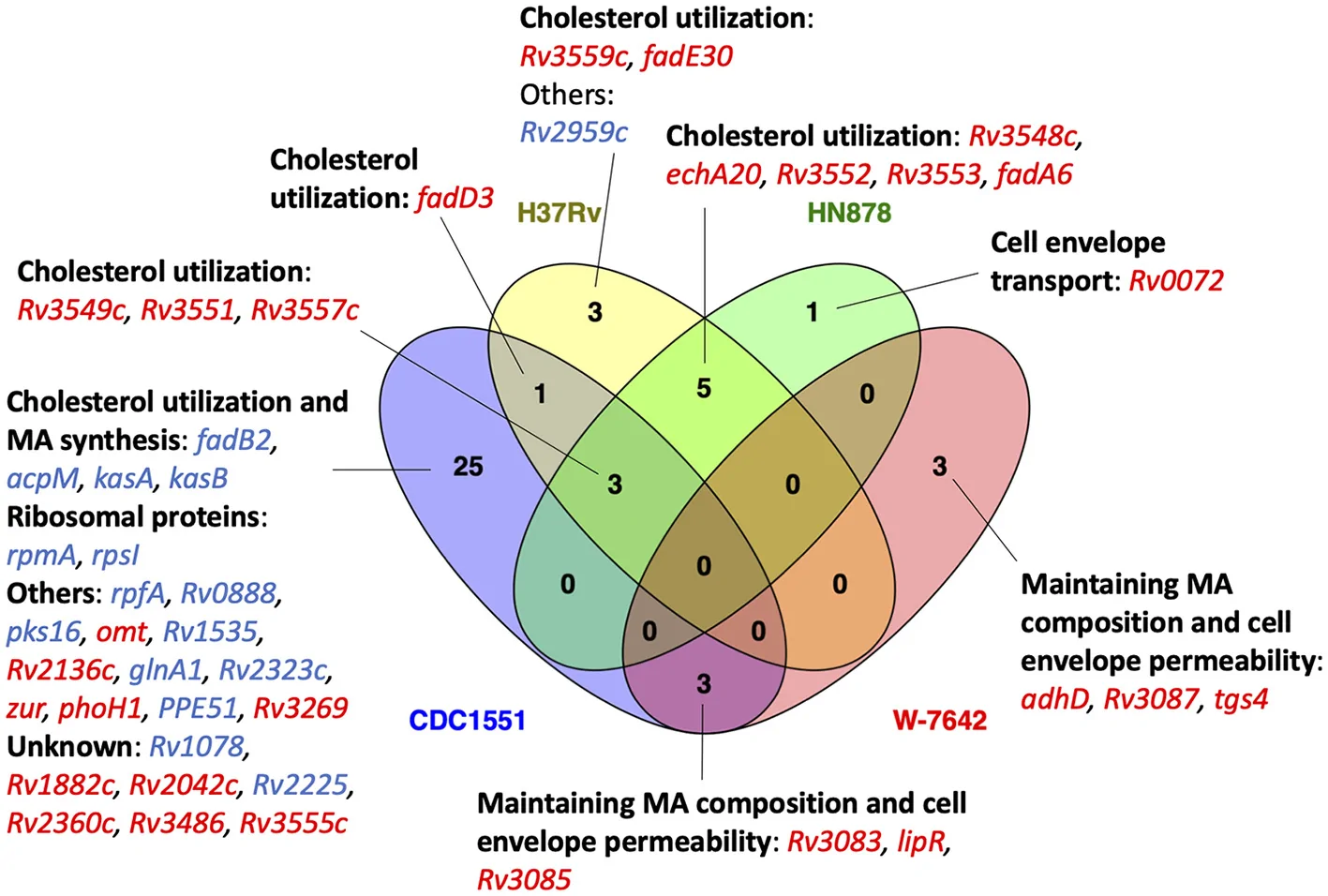
Venn diagram showing the number of shared (between 2 or more of the strains) and strain-specific DEGs (ALF-exposed *vs*. unexposed *M.tb*) after 15-min exposure. Upregulated DEGs are shown in red, and downregulated DEGs in blue. DEGs were grouped based on their function. Venn diagram was constructed using Venny 2.1.0 (53).

**Figure 7:**
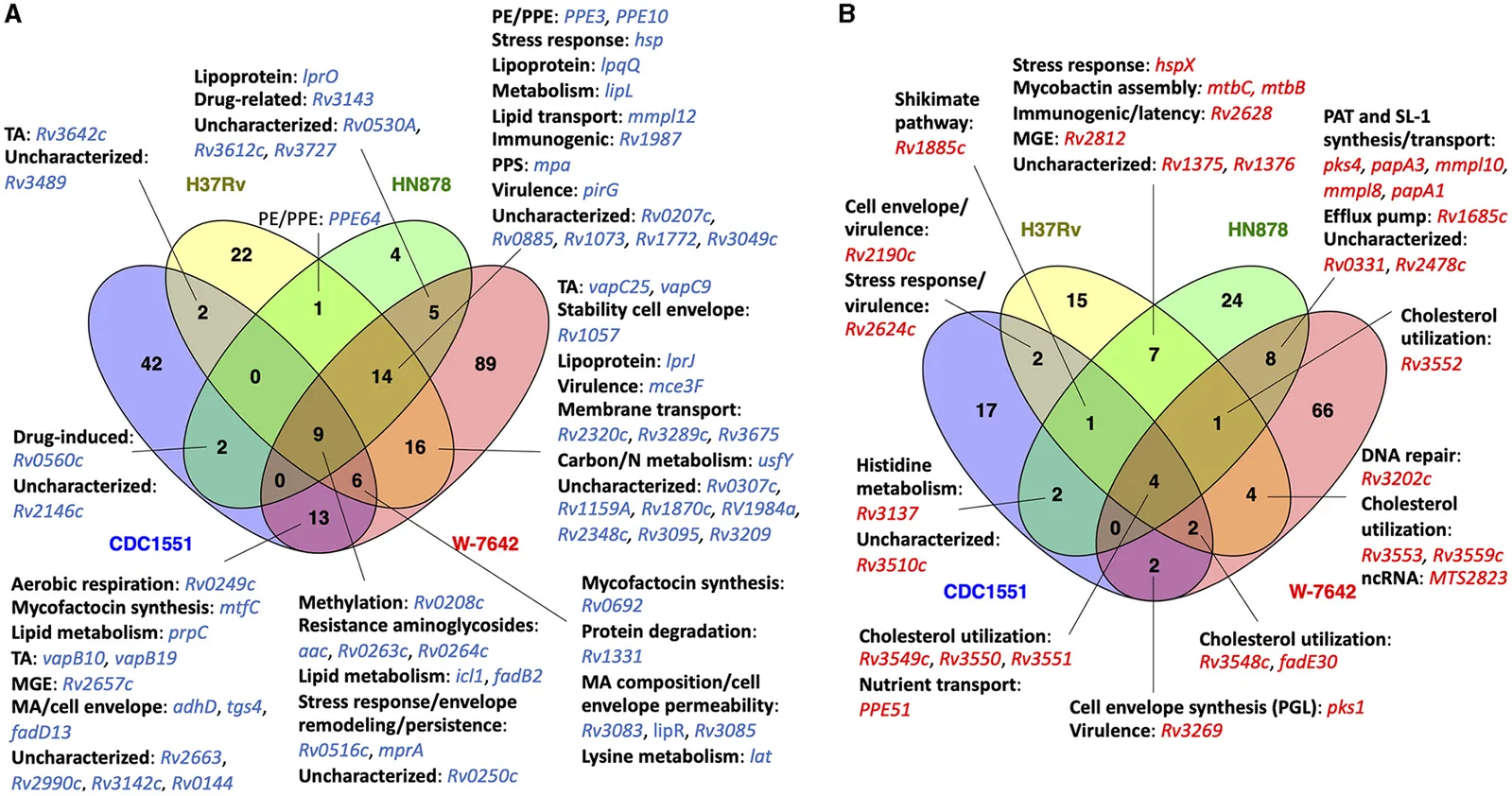
Venn diagram showing shared DEGs (ALF-exposed *vs*. unexposed *M.tb*) between two or more strains at 12-h post-ALF exposure: **(A)** downregulated genes (blue); **(B)** upregulated genes (red). DEGs were classified based on their function. Venn diagrams were constructed using Venny 2.1.0 (53).

In addition to the cholesterol utilization pathways, most *M.tb* strains showed upregulation of genes required for maintaining an appropriate mycolic acid layer composition and permeability of the *M.tb* cell envelope at 15 min post-ALF exposure, achieving statistical significance in strains CDC1551 and W-7642 (Figure 6, Supplementary Table S3). This included shared genes *Rv3083* (*mymA*), *Rv3084* (*lipR*), and *Rv3085* (*sadH*), as well as *Rv3086* (*adhD*), *Rv3087*, and *Rv3088* (*tgs4*), only significant in W-7642. In contrast, these genes were downregulated after 12 h of ALF exposure (Figure 7A). All these genes belong to the *mymA* operon, associated with maintaining the *M.tb* cell envelope under harsh conditions (44, 45).

At 12-h post-ALF exposure, we found several up and downregulated genes shared among two or more of the *M.tb* strains evaluated. Interestingly, both hypervirulent HN878 and drug resistant W-7642 showed significant upregulation of diacyltrehalose (DAT), penta-acyltrehalose (PAT) and sulfolipid-1 (SL-1) biosynthesis and transport genes *pks4, papA3, mmpL10, papA1*, and *mmpL8* (Figure 7B). These glycolipids are major *M.tb* cell envelope components (46), and some of them are significantly reduced on the *M.tb* cell envelope surface after exposure to human ALF (6), indicating a potential compensatory upregulation of these genes to restore the *M.tb* cell surface. Indeed, *pks1*, involved in the synthesis of immunomodulatory phenolic glycolipids (PGLs) produced by some *M.tb* strains and associated with cell envelope impermeability, phagocytosis, defense against oxidative stress and biofilm formation (46–48), was upregulated in all *M.tb* strains in this study, although was only significant in CDC1551 and W-7642 (Figure 7B, Supplemental Table S3). We note here, though, that many clinical isolates (including CDC1551), as well as laboratory strain H_37_R_v_, contain a *pks1-15* polymorphism associated with the inability of these strains to synthesize PGL (49).

*Rv1685c*, a TetR-like transcriptional regulator that controls an efflux pump with a potential role in the response to acid nitrosative stress (50), was also significantly expressed in HN878 and W-7642 (Figure 7B). Other upregulated genes at 12 h post-ALF exposure were associated to mycobactin assembly (*mtbB, mtbC*), involved in iron acquisition, growth in macrophages and virulence (51, 52), and stress response gene *hspX* in H_37_R_v_ and HN878, as well as potential immunogenic and virulence-associated genes (*Rv2190c, Rv2624c*) in CDC1551 and H_37_R_v_.

We also found several shared downregulated genes after 12-h of ALF exposure (Figure 7A). Genes associated with resistance to aminoglycosides (*acc, Rv0263c, Rv0264c*) (54), lipid metabolism (*icl1, fadB2*) (55), and osmotic stress response and persistence (*Rv0516c, mprA*) (56, 57) were consistently downregulated in all four *M.tb* strains. Further, fatty acid (FA) transporter *mmpL12* was down in all *M.tb* strains but CDC1551. We also observed downregulation of mycofactocin synthesis genes, which have a potential multifaceted role in alcohol metabolism of *M.tb*, hypoxia adaptation, and redox homeostasis (58), as well as several lipoproteins and toxin-antitoxin (TA) systems in most of the *M.tb* strains, among others (Figure 7A).

### Strain-specific transcriptional profiles after ALF exposure

In addition to shared transcriptional responses among *M.tb* strains due to ALF exposure, we also observed unique expression signatures attributed to the *M.tb* genomic background at both time points tested, shown in Supplementary Table S5. At 15-min post-ALF exposure, most of the strain-specific DEGs were still related to the KstR2 regulon and maintaining the mycolic acid layer composition and permeability of the *M.tb* cell envelope. We found a gene encoding a probable transmembrane protein responsible for the transport of glutamine across the membrane (*Rv0072*) significantly increased in the hypervirulent HN878 (log_2_FC >5). The highly transmissible CDC1551 showed the most changes at this time point, with 25 unique DEGs (Figure 6). It included downregulation of essential mycolic acid biosynthesis genes *acpM, kasA*, and *kasB* (59, 60), *pks16* involved in biofilm formation (61), and cell envelope-associated genes such as *rpfA* (stimulates resuscitation of dormant cells), *Rv0888* (induces formation of neutrophil extracellular traps or NETs) (62), and *glnA1* (regulates nitrogen levels during poly-L-glutamine or PLG synthesis in the cell envelope) (63, 64). Interestingly, a global transcriptional regulator (*Rv2359* or *zur*) that represses transcription of genes involved in zinc homeostasis was upregulated in CDC1551 (Figure 6).

At 12-h post-ALF exposure, CDC1551 downregulated several cell envelope genes, including *Rv0559c* and *phoY*, with suggested roles in drug resistance (65, 66). Sigma factor E (*sigE*), involved in persistence to anti-TB drugs and essential for virulence and phagosome maturation in macrophages, was downregulated (67, 68), as well as *msrB*, which encodes a repair enzyme for proteins that are inactivated by oxidation and reported as upregulated in the resuscitation of dormant *M.tb* (69). In addition, several PE/PPE genes, involved in virulence and the activation of proinflammatory responses, and stress-induced genes (e.g., *hspR, clpB*) were also downregulated. In contrast, only a few unique genes were upregulated in CDC1551, including *Rv2958c* and *Rv2959c*, involved in the glycosylation of cell envelope PGLs, and some fatty acid biosynthesis genes, which were initially downregulated at 15-min ALF post-exposure.

Interestingly, H_37_R_v_ showed upregulation of several latency-associated *dosR* regulon genes after 12-h of ALF exposure (*Rv2623* or *TB31.7, Rv2625c, hrp1*) (70), as well as *pncB2*, involved in NAD biosynthesis during host invasion that was found increased during hypoxia (71), indicating a potential role during persistence (Supplementary Table S5). Gene *irtB*, involved in iron homeostasis (72, 73), and a couple of genes associated with resistance to oxidative stress (*lpdA, Rv1050*) were also upregulated. Similar to CDC1551, we found two sigma factors with decreased expression (*sigB* and *sigD*), potentially involved in the regulation of the stationary phase of *M.tb* (74). Other unique downregulated genes included *pup*, with a role in proteasomal degradation, and TA-associated genes (Supplementary Table S5).

Of the 28 unique DEGs found in hypervirulent HN878 at 12-h post-ALF exposure, 24 showed significant increased expression (log_2_FC >1 and FDR <0.1) (Supplementary Table S5). Within the cell envelope functional category, we found *esxI* upregulated, which encodes an ESAT-6 protein secreted by the ESX-5 system that is involved in the virulence of *M.tb* (75). We also found upregulated the lipoprotein *lppJ* of unknown function, and the cluster *Rv1686c/Rv1687c*, a putative efflux pump responsible for the transport of undetermined substrates (potentially drugs) across the membrane that is activated under several environmental stressors (76, 77). Further, unique DEGs from the intermediate metabolism and respiration category were all upregulated in HN878, including *glbN* that encodes an oxygen transport protein upregulated during macrophage infection (78), molybdenum cofactor biosynthesis protein MoaA1, strongly induced under hypoxia (79), and *dxs2*, involved in the mycobacterial ability to prevent acidification of the phagosome (80) (Supplementary Table S5). Contrary to CDC1551 and H_37_R_v_, in HN878 two PE/PPE genes showed increased expression. The only unique downregulated genes in HN878 were two transmembrane proteins of unknown function, an uncharacterized hypothetical protein, and *fadE8*, involved in lipid degradation (Supplementary Table S5).

Finally, MDR-*M.tb* W-7642 showed the most strain-specific DEGs (#155) after 12-h post-ALF exposure compared to unexposed bacteria, with 66 upregulated and 89 downregulated genes (Figure 7). We found upregulation of several genes associated with the ESX-3 system and other genes related to iron acquisition and utilization (*esxH, eccD3, mycP3, Rv0285* or *PE5, hemE, hemL, Rv2047c*), essential for mycobacterial growth *in vitro* and virulence (81) (Supplementary Table S5). In contrast, ESX-1 genes *espD, espC*, and *espA* were downregulated. The ESX-1 system secretes host membrane-targeting proteins such as ESAT-6 that interrupt the innate immune response against *M.tb* (82, 83). Other ESAT-6-like proteins were found downregulated (EsxJ, EsxP, EsxD) in W-7642, with the exception of EsxL that was upregulated. Overall, genes generally induced during starvation, persistence, and/or during the stationary phase of *M.tb* showed decreased expression (e.g., *rsbW, vapC44, mce1R, lipU, Rv3241c*) (84), as well as sigma factors *sigF* and *sigI* (Supplementary Table S5). However, we observed increased expression of *M.tb* cell envelope biosynthesis genes and lipid metabolism (*Rv2974c, aftD, murE, murF, Rv3807c, accD6, Rv3031, kasB, acpS, fas, ppsC, ppsD, pks15*), amino acid metabolism (*leuC, leuD, argD, trpA*), and DNA repair genes (*ruvB, Rv3201c, Rv3433c, radA*). Interestingly, W-7642 was the only strain with decreased expression of *ethA* (85), but increased expression of *Rv2989* (86), both associated with drug resistance. Other unique DEGs in strain W-7642 are shown in Supplementary Table S5.

RNA-seq results for selected genes of interest were validated by qPCR, showing similar fold change values (Supplementary Figure S4).

## Discussion

Our published studies on the role of ALF during *M.tb* infection indicate that human ALF hydrolases alter the *M.tb* cell envelope, with an impact in subsequent interactions with immune host cells *in vitro* and *in vivo* (6–12). This suggests that physiological levels of ALF play an important role in shaping the *M.tb* cell envelope during the very early stages of infection. Here, we used RNA-seq to depict the transcriptional responses to ALF exposure of four *M.tb* strains with different degrees of transmissibility, virulence, and drug resistance. Our results define for the first time the overall impact of human ALF, first lung component encountered by *M.tb* during infection, on the adaptation of this pathogen to the human alveolar space. We also determined whether exposure to ALF has different transcriptional effects based on the *M.tb* genomic background, which might further define phenotypic differences among *M.tb* strains. Indeed, we observed temporal shared and strain-specific transcriptional responses in *M.tb* after exposure to human ALF, demonstrating that *M.tb* can metabolically adapt to the lung mucosa prior to infecting host cells.

The most striking effect of ALF exposure in the majority of *M.tb* strains studied was the activation of cholesterol utilization pathways, specifically the KstR2 regulon, with some genes induced at 15-min post-ALF exposure and maintained at 12-h (Figure 5). Conversely, expression of genes under the KstR1 regulon remained practically unaltered after exposure to ALF, as well as most of the genes belonging to the Mce3R and SigE regulons (Supplementary Table S3). The ability of *M.tb* to utilize host-derived cholesterol as a source of carbon is essential for survival of *M.tb* in the human host, especially in the nutrient-deprived macrophage environment, where cholesterol significantly contributes to *M.tb* intracellular growth and pathogenesis (37, 87). Indeed, metabolites originated from cholesterol degradation such as pyruvate, acetyl-CoA or succinyl-CoA, can be incorporated into *M.tb* metabolic pathways for cell envelope lipid biosynthesis and production of energy. Further, *M.tb* uses a cholesterol-dependent pathway to infect host cells, induces a foamy macrophage phenotype, and extracts cholesterol and lipids from caseous granulomas to persist, demonstrating the importance of host cholesterol utilization during both early and latent stages of the infection (88, 89). Importantly, deletion of genes associated with cholesterol catabolism result in attenuated *M.tb* strains (38, 90). Our transcriptomics data suggest that the metabolic adaptation of *M.tb* to use host cholesterol is initiated even before its intracellular stage in host cells, where extracellular *M.tb* starts consuming host cholesterol in the ALF, activating cholesterol degradation pathways that will later be key for infection and survival within host cells. Indeed, cholesterol constitutes most of the neutral lipid in lung surfactant, and levels vary in response to a number of physiological and pathophysiological factors (91–93).

Further, most *M.tb* strains exposed to ALF showed increased expression of some of the genes in the *mymA* operon (*Rv3083-Rv3089*, Supplementary Table S3) after 15-min of contact with ALF (Figure 6). This operon is induced when *M.tb* is exposed to acidic pH and during macrophage infection, demonstrating a role in the modification of fatty acids required for maintaining the appropriate permeability of the cell envelope and mycolic acid layer composition under harsh conditions (44, 45). Furthermore, *mymA* mutants are more susceptible to known anti-TB drugs and detergents, and infection with those mutants results in decreased bacterial load in the spleen of guinea pigs, showing that this operon is also important for *M.tb* pathogenesis and drug resistance (94). This could explain why MDR W-7642 was the only strain in our dataset that had significantly increased expression of all genes from the *mymA* operon after 15-min exposure to ALF. In contrast, *mymA* genes were downregulated at 12-h post-ALF exposure (Figure 7A). These results suggest that, early after contact, *M.tb* upregulates genes that help maintain the cell envelope integrity to compensate for the changes generated upon contact with ALF, but this effect is only temporal and expression of these genes is decreased after a certain period, maybe due to a certain degree of *M.tb* adaptation to the ALF environment. What we known is that ALF exposure to *M.tb* does not affect bacterial viability despite the reported cell envelope (6, 7) and metabolic changes (this study).

In this context, we also found changes in the expression of genes associated to cell envelope biosynthesis and remodeling in ALF-exposed *M.tb*. In fact, we previously reported that *M.tb* exposure to human ALF alters the *M.tb* cell envelope by significantly reducing the total amount of two major *M.tb* cell surface virulence factors, the mannose-capped lipoarabinomannan (ManLAM) and the trehalose dimycolate (TDM), both implicated in promoting phagosome maturation arrest and *M.tb* intracellular growth (5, 6, 46). In response to this reduction, we found in a previous study that *M.tb* Erdman (lineage 4) exposed to ALF upregulated genes involved in the synthesis of TDM and mannose-containing biomolecules (25). We did not observe these same transcriptional effects in ManLAM and TDM in this study, probably due to the fact that different *M.tb* strains were used. However, other cell envelope biosynthesis genes were upregulated by ALF exposure. Indeed, hypervirulent HN878 and MDR W-7642 strains from lineage 2 (Beijing) showed significantly increased expression of DAT/PAT, and SL-1 biosynthesis and transport genes, including MmpL proteins, important for the physiology and pathogenesis of *M.tb* (Figure 7B) (95). Together with ManLAM and TDM, these cell envelope glycolipids have the ability to modulate host cell immune responses (96), as well as blocking phagosome maturation (e.g., fusion with lysosomes) in infected phagocytes (97, 98), supporting the hypervirulent phenotype found in most Beijing lineage strains.

In addition, other genes involved in the biosynthesis of the *M.tb* cell envelope were significantly upregulated in W-7642, such as *pks1* and *pks15* (PGL synthesis) (48, 49), *murE* and *murF* (peptidoglycan synthesis) (99, 100), *aftD* (biosynthesis of the arabinogalactan region of the *M.tb* cell envelope backbone known as mycolyl-arabinogalactan-peptidoglycan complex or mAGP) (101), *Rv2974c* (hypothetical protein potentially involved in glycerolipid and glycerophospholipid metabolism) (102), and several lipid and amino acid metabolic genes implicated in cell envelope biomass synthesis (99) (Supplementary Table S3). These results indicate that, even though exposure to functional ALF cleaves components of the cell envelope (6), strains HN878 and W-7642 are capable of reprograming their metabolism and upregulate genes involved in the synthesis of cell surface components involved in *M.tb* survival and pathogenesis (DAT/PAT and SL-1), suggesting a potential lineage-specific response to ALF.

Conversely, other cell envelope-associated genes were significantly downregulated after exposure to ALF for 12-h, including protein export genes in CDC1551 (Supplementary Table S3) and lipoproteins in H_37_R_v_, HN878, and especially W-7642. Lipoproteins are membrane-anchored proteins involved in virulence and immunity in *M.tb*, with a dual role, some promote survival of *M.tb* while others induce host protective immune responses (103). The ones found here are some of the less studied, and their specific function/s are still unknown. Interestingly, W-7642 was the only strain with decreased expression of *ethA*, which encodes the enzyme responsible for the activation of the pro-drug ethionamide (85, 104), but increased expression of *Rv2989*, an IclR family transcriptional regulator involved in increased isoniazid tolerance (86). Thus, exposure to ALF might be promoting the drug resistance ability of W-7642, which remains to be elucidated.

ESX or type VII secretion systems, responsible for the secretion of *M.tb* proteins to evade host immune responses (105), were also impacted after 12-h of ALF exposure, especially in MDR W-7642. In this strain, we found significantly increased expression of ESX-3 genes, involved in iron and nutrient uptake and utilization (106), as well as other iron homeostasis genes (e.g., *mtbB, mtbC*) important for intracellular growth that were also upregulated in the rest of the strains. In contrast, genes from the ESX-1 system, involved in the lysis of the phagosomal membrane and phagosomal escape of the bacteria (107), were downregulated. Increase of ESX-3 and other iron homeostasis genes after human ALF exposure could be preparing *M.tb* for subsequent infection of macrophages by improving its ability to survive and grow in phagosomes in detriment of other functions (e.g., the ability to escape the phagolysosome). Associated with the ESX-5 secretion system, gene *esxI* (108), suggested to modulate growth in intracellular bacteria, was uniquely upregulated in HN878, potentially related to its hypervirulent genotype.

Several sigma factors (*sigB, sigD, sigE, sigF, sigI*) were found downregulated after ALF exposure in all *M.tb* strains studied (significant in all but HN878). Sigma factors are known to contribute to *M.tb* pathogenesis by regulating the expression of genes involved in the adaptation of *M.tb* to stress conditions encountered during different stages of infection (109), including the stationary phase. Accordingly, the majority of stress response DEGs detected, as well as genes associated to hypoxia and latency, were downregulated in most strains. H_37_R_v_ was the exception, showing upregulation of genes with potential roles during persistence and oxidative stress (e.g., *TB31.7, hrp1, hspX, pncB2, lpdA*) after exposure to ALF for 12-h (Supplementary Table S5). Overall, this suggests that exposure to ALF might be reducing the ability of some *M.tb* strains (CDC1551, HN878, W-7642) to adapt to long-term survival and persist in a latent state, which remains to be investigated.

Finally, all DEGs from the PE/PPE functional category were downregulated in both CDC1551 and H_37_R_v_ after 12-h of exposure to ALF, while some were upregulated in HN878 and W-7642 (Beijing lineage strains) (Supplementary Table S3). While most PE/PPE proteins are still uncharacterized and their molecular mechanisms are unknown, it is suggested that these are involved in *M.tb* virulence and modulation of host immune responses (110). The fact that after exposure to ALF some of these are upregulated only in Beijing lineage strains could indicate a potential adaptive advantage of HN878 and W-7642 to the human host, and suggests again a potential lineage-specific response to ALF. Conversely, TA systems, associated to *M.tb* pathogenicity and persistence (111), were downregulated in most of the *M.tb* strains at 12 h post-ALF exposure, which might be explained by the ALF trying to induce a protective response (6).

In summary, this study shows that human ALF changes the metabolism of *M.tb* in a temporal and strain- and/or lineage-specific manner, and that it is an important environment to consider when studying *M.tb* pathogenesis. Most of the effects were observed at 12-h ALF post-exposure, with the exception of cholesterol utilization pathways that were rapidly upregulated starting at 15-min after exposure. Early exposure (15-min) to ALF also drives upregulation of genes associated with maintaining the appropriate composition of the cell envelope. In addition, our transcriptional analyses show remodeling of the *M.tb* cell envelope, upregulation of genes associated with nutrient and iron uptake (e.g., ESX-3), and downregulation of sigma factors and other genes associated with persistence and pathogenesis. These results indicate that the ALF environment promotes changes in *M.tb*'s metabolism, preparing the bacteria for its encounter with host cells (e.g., by upregulating cholesterol utilization pathways and iron/nutrient uptake genes, key for intracellular survival), while at the same time *M.tb* is trying to compensate for ALF-driven changes on the cell envelope early after exposure. In addition, we observed significant differences within strains upon human ALF exposure. Indeed, Beijing lineage strains HN878 and W-7642 upregulated genes associated with the synthesis of cell envelope glycolipids, potentially linked to their hypervirulent phenotype (22, 112).

A limitation of the study is the use of only two replicates from each *M.tb* strain and condition studied for the RNA-seq analysis. Increasing the number of replicates might increase the number of DEGs, especially at 15-min, further confirming our results. However, each replicate consisted of exposing each *M.tb* strain to a pool of 17 different human ALFs, providing us with a good representation of the ALF environment present in healthy participants. We opted for this strategy because using a pool instead of individual ALFs allows us to reduce human variability and focus on the overall/global effects of ALF exposure in each *M.tb* genotype rather than specific donor ALF-driven changes. Still, we understand that human variability strongly influences the outcomes of the *M.tb* infection and thus, the impact of individual ALFs on *M.tb* metabolism will be further investigated in future studies. In addition, ALF exposure times were selected based on our published data, where changes in the *M.tb* cell envelope were observed in as little as 15-min, with more prominent effects at 12-h (6, 25). Nonetheless, we note that these are not necessarily the most biologically relevant exposure times, since it is currently unknown for how long *M.tb* is in contact with ALF prior to and after infection of alveolar resident and/or compartment cells. What is known is that the recycling of ALF in the alveolar space is every 18 h (26). Thus, future studies will confirm *M.tb* gene expression profiles after longer ALF contact times.

Further, whether the transcriptional changes observed here are strain- and/or lineage-specific remains to be elucidated. This will be further investigated by including genetically close strains from the same lineages (2 and 4) as well as strains from other lineages. Ongoing and future studies will also define the short- and long-term effects of *M.tb* exposure to human ALF *in vitro* and *in vivo*. Regarding the RNA-seq analyses, we decided to use a less stringent FDR value of <0.1 to capture ALF-driven DEGs that would have been missed otherwise due to only having two replicates/condition, thus increasing the % of potential false positives. Finally, we realize that the metabolic status of *M.tb* grown on agar plates (this study) is unrelated to that of transmitted bacteria. Still, we consider that addressing how *M.tb* adapts metabolically to the first lung environment encountered (ALF) can provide valuable information on the early events of *M.tb* infection, which is still unknown and of major importance in the TB field.

This study provides new insights into specific *M.tb* metabolic pathways affected by exposure to human ALF and further defines transcriptional differences among *M.tb* strains exposed to ALF, highlighting the importance of the alveolar environment and its interactions with *M.tb* during the early infection process, potentially playing a major role in *M.tb* infection and pathogenesis outcomes.

## Data availability statement

All raw and processed sequencing data generated in this study can be found in the NCBI Gene Expression Omnibus (GEO; https://www.ncbi.nlm.nih.gov/geo/) (113) under accession number GSE228998.

## Ethics statement

The studies involving humans were approved by the Ohio State University Human Subjects IRB numbers 2008H0135 & 2008H0119 and Texas Biomedical Research Institute/UT-Health San Antonio Human Subjects IRB numbers HSC20170667H & HSC20170315H. The studies were conducted in accordance with the local legislation and institutional requirements. The participants provided their written informed consent to participate in this study.

## Author contributions

AA-G: Conceptualization, Data curation, Formal analysis, Funding acquisition, Investigation, Methodology, Project administration, Supervision, Validation, Visualization, Writing—original draft, Writing—review & editing. AG-V: Formal analysis, Investigation, Visualization, Writing—review & editing. AS: Investigation, Writing—review & editing. AO-F: Investigation, Writing—review & editing. AH: Investigation, Writing—review & editing. JP: Resources, Writing—review & editing. DM: Resources, Writing—review & editing. MW: Resources, Writing—review & editing. YW: Data curation, Writing—review & editing. JT: Conceptualization, Funding acquisition, Methodology, Project administration, Resources, Supervision, Writing—original draft, Writing—review & editing.
