# Supplementary material for: Exposure of Mycobacterium tuberculosis to human alveolar lining fluid shows temporal and strain-specific adaptation to the lung environment
Source: Front Tuberc. 2024 Mar 4;2:1352806. doi: 10.3389/ftubr.2024.1352806 (PMC13494945; doi:10.3389/ftubr.2024.1352806)
Supplement: Supplementary file 1 [file Image_1.PDF]

Supplemental Figure S1

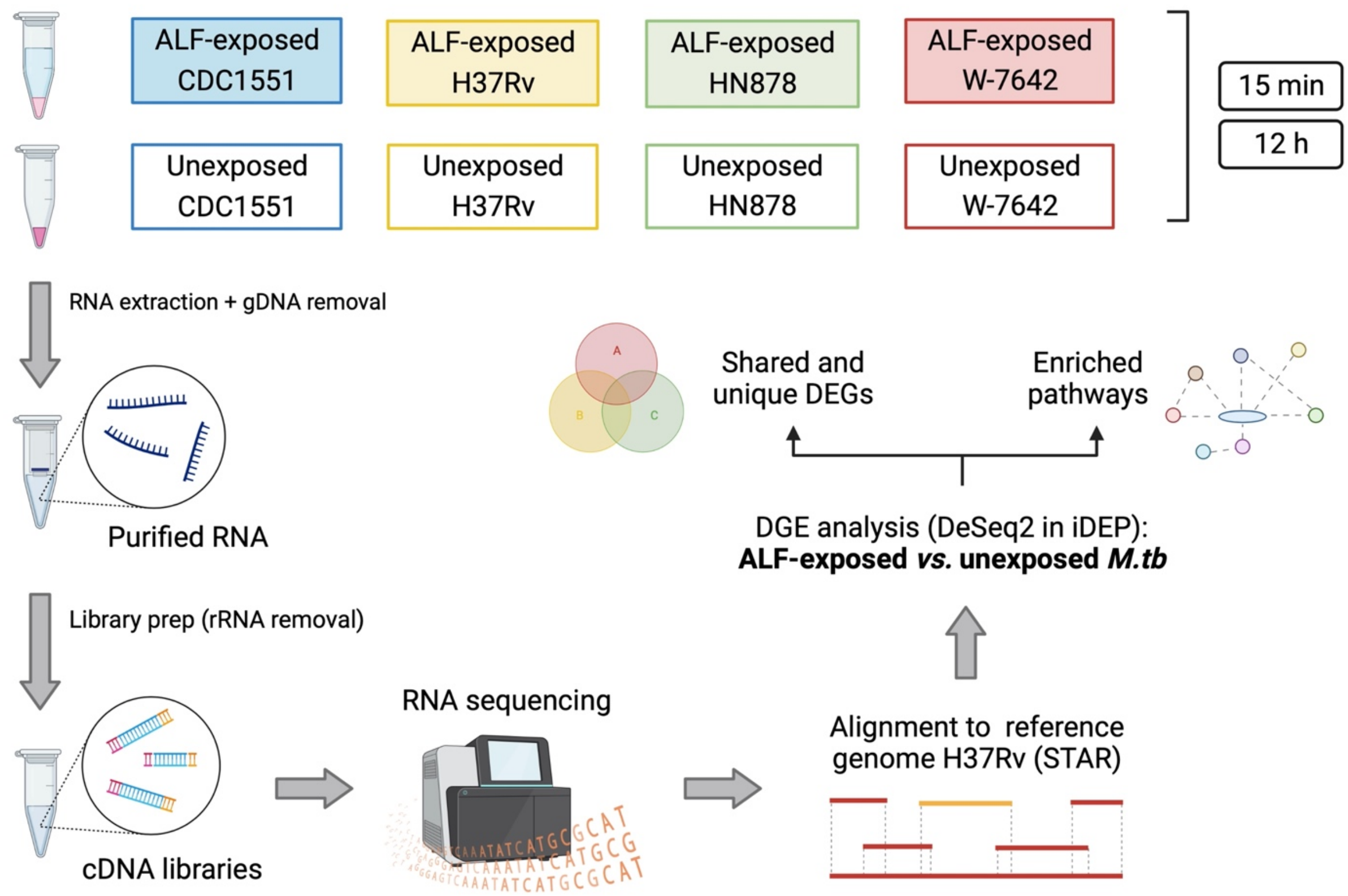

Supplemental Figure S1. Diagram of experimental conditions and strategy for RNA-seq and data analyses.

Supplemental Figure S2

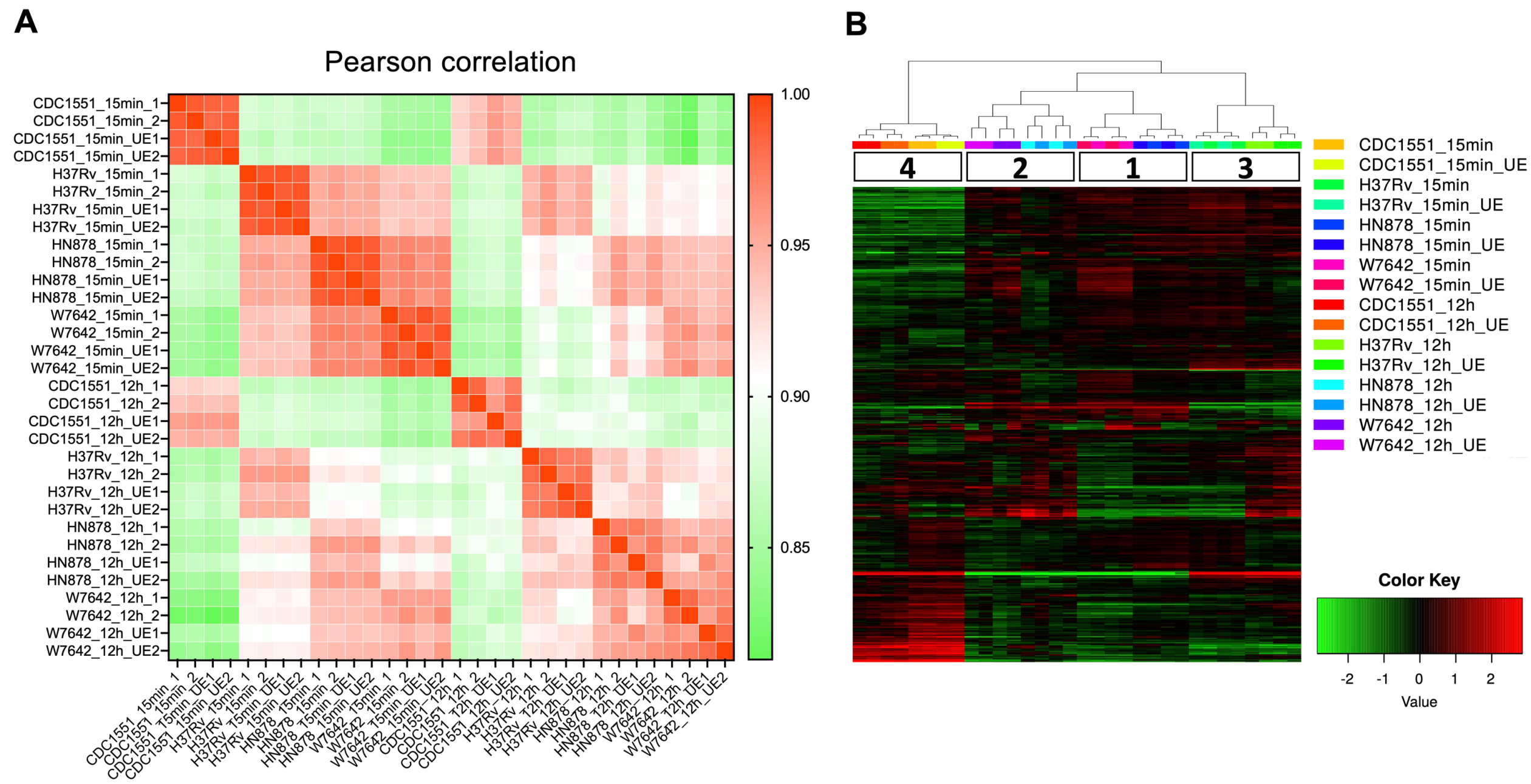

Supplemental Figure S2. **(A)** Pearson correlation calculated in iDEP.94 and represented as a heatmap. Biological replicates are shown as 1 and 2; UE (unexposed *M.tb*), otherwise indicates ALF-exposed bacteria; **(B)** Heatmap of normalized gene expression (red: upregulated, green: downregulated) with hierarchical clustering of samples analyzed in this study: four *M.tb* strains (CDC1551, H$_{37}$R$_{v}$, HN878, W-7642), exposed to a human ALF pool or unexposed (UE) for 15-min or 12-h. Replicates (n=2) are shown in the same color. Graph was generated in iDEP.94.

Supplemental Figure S3

A

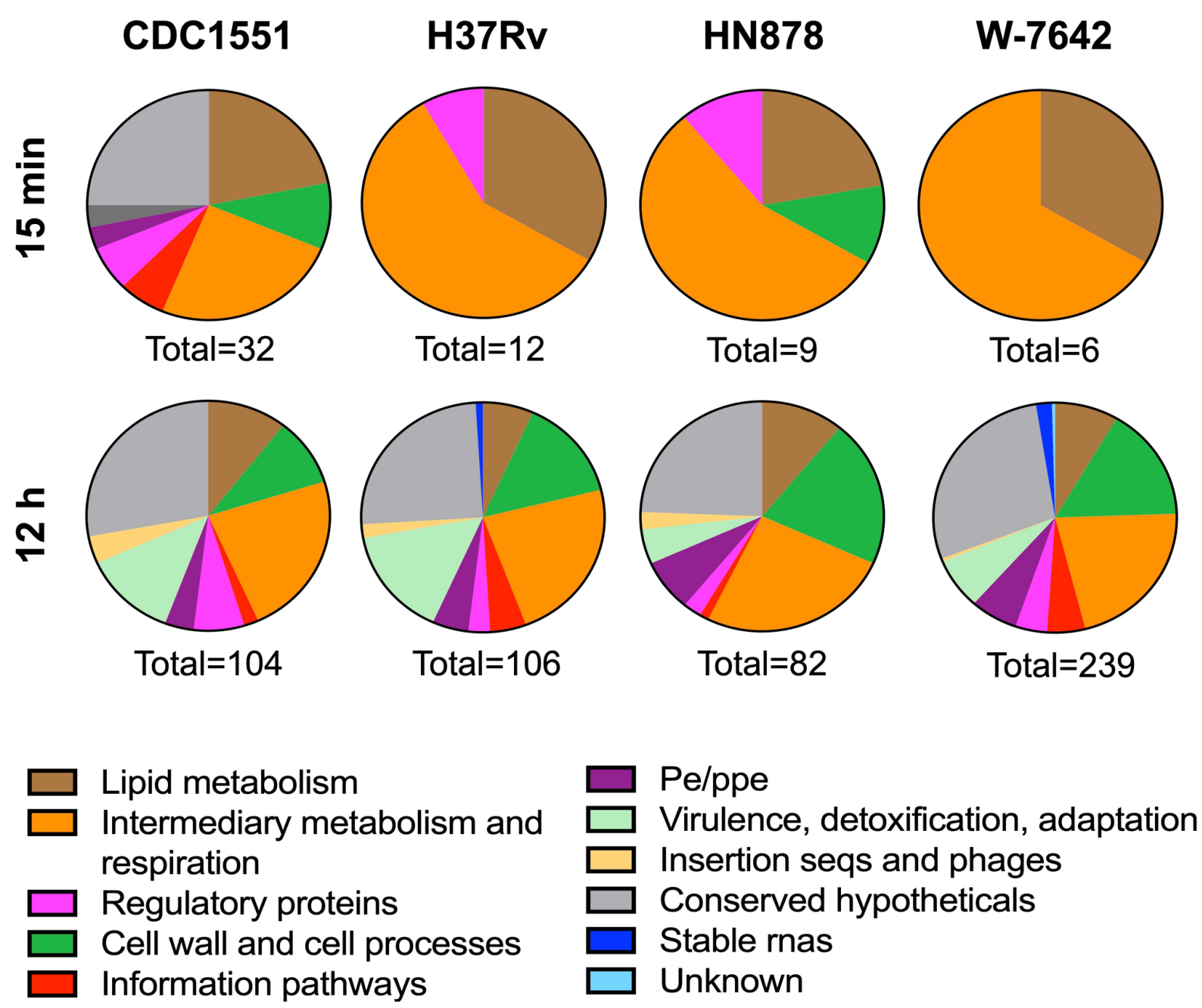

B

	15 min				12 h			
	CDC1551 n (%)	H37Rv n (%)	HN878 n (%)	W-7642 n (%)	CDC1551 n (%)	H37Rv n (%)	HN878 n (%)	W-7642 n (%)
Lipid metabolism	7 (21.9)	4 (33.3.)	2 (22.2)	2 (33.3)	11 (10.6)	7 (6.7)	9 (11)	20 (8.4)
Cell wall and cell processes	3 (9.4)	0 (0.0)	1 (11.1)	0 (0.0)	10 (9.6)	15 (14.4)	17 (20.7)	38 (16)
Intermediary metabolism and respiration	8 (25)	7 (58)	5 (55.6)	4 (66.7)	24 (23.1)	24 (23.1)	21 (25.6)	51 (21.5)
Information pathways	2 (6.3)	0 (0.0)	0 (0.0)	0 (0.0)	2 (1.9)	5 (4.8)	1 (1.2)	12 (5.1)
Regulatory proteins	2 (6.3)	1 (8.3)	1 (11.1)	0 (0.0)	7 (6.7)	3 (2.9)	2 (2.4)	10 (4.2)
Pe/ppe	1 (3.1)	0 (0.0)	0 (0.0)	0 (0.0)	4 (3.8)	5 (4.8)	6 (7.3)	15 (6.3)
Virulence, detoxification, adaptation	2 (3.1)	0 (0.0)	0 (0.0)	0 (0.0)	13 (12.5)	16 (15.4)	4 (4.9)	17 (7.1)
Insertion seqs and phages	0 (0.0)	0 (0.0)	0 (0.0)	0 (0.0)	4 (3.8)	2 (1.9)	2 (2.4)	1 (0.42)
Conserved hypotheticals	8 (25)	0 (0.0)	0 (0.0)	0 (0.0)	29 (27.9)	26 (25)	20 (24.4)	67 (28.3)
Stable rnas	0 (0.0)	0 (0.0)	0 (0.0)	0 (0.0)	0 (0.0)	1 (0.9)	0 (0.0)	5 (2.1)
Unknown	0 (0.0)	0 (0.0)	0 (0.0)	0 (0.0)	0 (0.0)	1 (0.0)	0 (0.0)	1 (0.42)
**TOTAL**	32 (100)	12 (100)	9 (100)	6 (100)	104 (100)	106 (100)	82 (100)	239 (100)

Supplemental figure S3. **(A)** Distribution of functional categories (as defined in Mycobrowser) of DEGs for each strain and ALF exposure time. Pie chart was generated in Graphpad Prism v9.1.1. **(B)** Total number (n) and percentage (%) of DEGs for each functional category.

Supplemental Figure S4

A

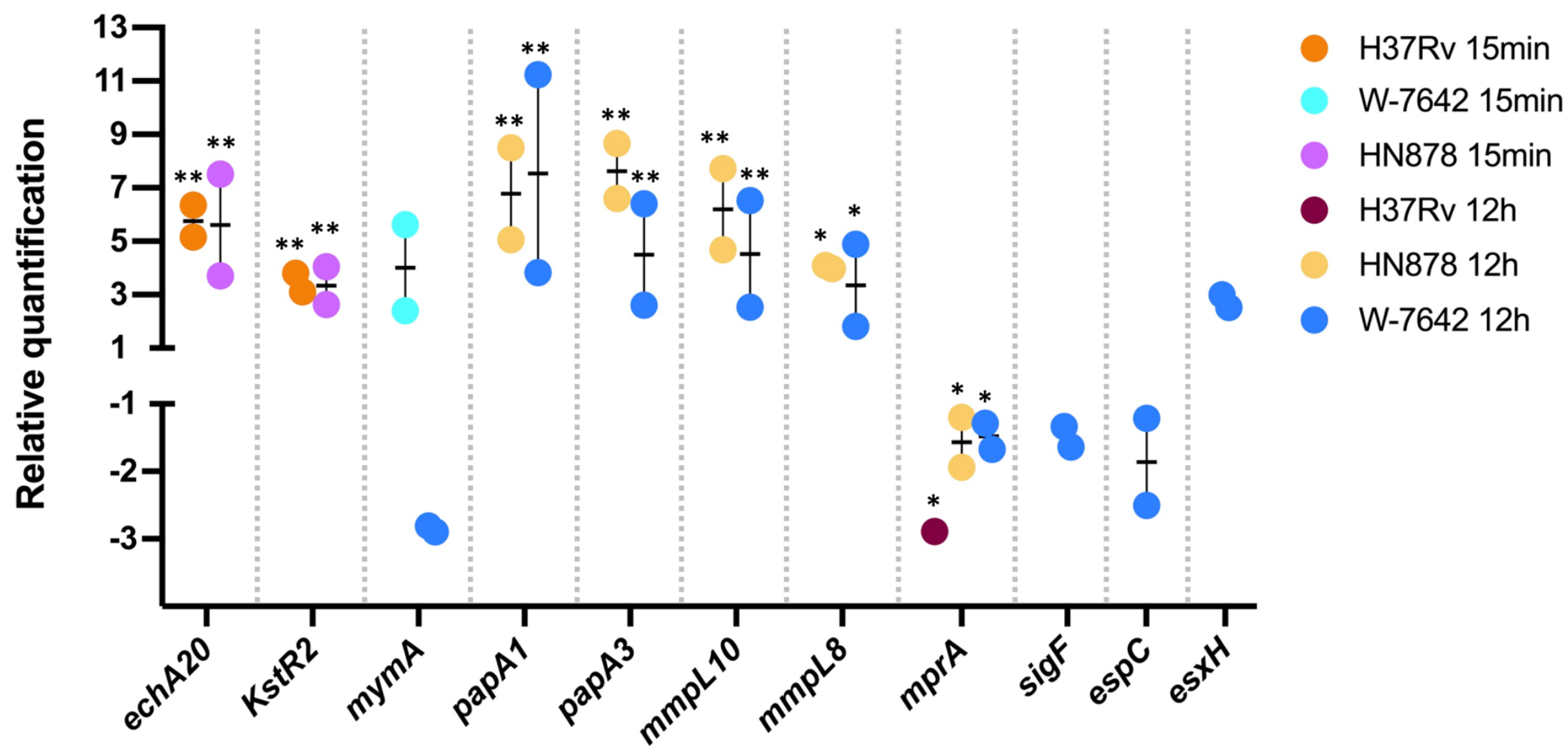

B

	H37Rv 15min		HN878 15min		W-7642 15min		H37Rv 12h		HN878 12h		W-7642 12h	
	qPCR	RNAseq	qPCR	RNAseq	qPCR	RNAseq	qPCR	RNAseq	qPCR	RNAseq	qPCR	RNAseq
echA20	5.75	4.68	5.61	4.20								
KstR2	3.45	3.05	3.34	3.00								
mymA					4.01	3.52					-2.85	-2.69
papA1									6.78	4.57	7.53	4.63
papA3									7.63	4.44	4.50	3.10
mmpL10									6.20	3.66	4.53	3.06
mmpL8									4.04	3.32	3.35	3.20
mprA							-2.89	-2.74	-1.57	-2.17	-1.48	-3.43
sigF											-1.49	-2.48
espC											-1.86	-2.58
esxH											2.76	2.07

Supplemental figure S4. Validation of gene expression by RT-qPCR for selected targets and strains. **(A)** RT-qPCR was performed as described in the methods and relative quantification was calculated using the $2^{-\Delta\Delta CT}$ method with *sigA* as the reference gene. Values are expressed as positive or negative fold changes (ALF-exposed *vs.* unexposed *M.tb*,) and plotted using Graphpad Prism v9.1.1. Statistical analysis (ΔCt ALF-exposed *vs.* ΔCt unexposed *M.tb* for each of the strains and timepoints): 2-way ANOVA for multiple comparisons with an uncorrected Fisher's LSD test; * *p*-value < 0.05; ** *p*-value < 0.005. Two dots of the same color represent biological replicates. **(B)** Comparison of fold changes (n=2 replicates) in qPCR and RNA-seq data for selected genes (ALF-exposed *vs.* unexposed *M.tb*). Positive and negative Log_2FC in RNA-seq data were converted to FC. Upregulated and downregulated genes are shown in yellow and blue, respectively.
